# Amino Acid-Enriched Formula for the Post-Operative Care of Extraction Sockets Evaluated by 3-D Intraoral Scanning

**DOI:** 10.3390/ijerph19063302

**Published:** 2022-03-11

**Authors:** Saverio Cosola, Giacomo Oldoini, Michela Boccuzzi, Enrica Giammarinaro, Annamaria Genovesi, Ugo Covani, Simone Marconcini

**Affiliations:** 1 Department of Stomatology, Tuscan Stomatologic Institute, Foundation for Dental Clinic, Research and Continuing Education, 55041 Camaiore, Italy; s.cosola@hotmail.it (S.C.); giacomo.oldoini88@gmail.com (G.O.); e.giammarinaro@gmail.com (E.G.); anm.genovesi@gmail.com (A.G.); covani@covani.it (U.C.); simosurg@gmail.com (S.M.); 2 Department of Dentistry, Unicamillus International Medical University, 00100 Rome, Italy

**Keywords:** hyaluronic acids, extraction surgery, amino acids, Aminogam, wound healing

## Abstract

Background: Hyaluronic acid and amino acids play an important role in the wound healing process, stimulating the development of the connective tissue and the activity and proliferation of fibroblasts. The aim of the present controlled clinical study was to evaluate the clinical efficacy of a topical gel formula containing hyaluronic acid and amino acids in terms of wound closure rate, painkiller intake, and patients’ reported pain and edema. Methods: This study included patients in need of a single tooth extraction. Patients were randomized into two groups with differing post-operative care regimens. Patients in the test group used the amino acid and hyaluronic acid-based gel, while the control group did not use any product. Each parameter was measured in both groups at different time points: immediately after surgery, and after 7, 14, 30, and 60 days. Results: A total of 40 patients (46.52 ± 9.84 years old) completed the observational period, and 40 extraction sockets were examined. After 7 days, the edema was significantly lower in the test group. The reported pain was lower in the test group without a significant difference, except for the first time point at 7 days. With the follow-up questionnaire, patients declared to have taken painkillers mainly during the first 7 days after surgery; however, the test group showed a lower need for painkillers than the control group. Conclusion: The post-operative and domiciliary use of an amino acid and hyaluronic acid-based gel for the management of soft tissue closure after tooth extraction is a valid coadjutant to reduce swelling, pain, and the need for painkillers. Additional studies are required to support the results of the present study.

## 1. Introduction

Patient-related outcomes (PROMs) represent a major issue when it comes to oral surgery. A fast healing of the surgical wound and the patient’s comfort after surgery are part of the modern definition of clinical success for minor surgeries, such as routine non-complicated tooth extraction. Surgical trauma causes inflammatory sequelae, which can be considered as a physiological primitive defense mechanism aimed at restoring the integrity of the tissues. The healing response consist of hemostasis, inflammation, proliferation, and wound remodeling. Wound healing is a dynamic process which begins with vasoconstriction and blood clot formation, which represents a reservoir for the cytokines and the grow factors for the proliferative phase [1,2].

During the repair process, proinflammatory cytokines, chemokines, and prostaglandins, such as tumor necrosis factor α (TNF-α) and interleukin-1β (IL-1β), are released and evoke pain via the direct activation and sensitization of nociceptors [3].

The remodeling phase is particularly important in the context of any future prosthetic rehabilitation. The main feature of this phase is the deposition of collagen in an organized and well-mannered network [4].

HA is a hygroscopic macromolecule and is highly osmotic. Within the skin and the oral mucosa, this property is likely to be relevant in controlling tissue hydration during periods of change, such as in the inflammatory process or with the response to tissue injury.

Thus, HA might help the healing phases and reduce the level of inflammation in the context of oral surgeries [5,6]. The anti-inflammatory effect may be due to the action of exogenous HA as a scavenger to drain prostaglandins, metalloproteinases, and other bio-active molecules [7]. The antiedematous effect may also be related to the osmotic activity [8].

The local application of hyaluronic acids and amino acids (glycine, L-lysine, L-leucine, L-proline) could locally influence the healing phases by stimulating fibroblast proliferation and indirectly enhancing the biosynthesis of collagen. These reactions promote the angiogenesis and the proliferation of keratinocytes, so the epithelialization is reported to be faster, and the surgical wound will heal quicker [9,10].

Additionally, some authors reported that hyaluronic acid has a role in bone healing by facilitating cell migration, proliferation, and differentiation [11].

The aim of the present study was to evaluate the clinical efficacy and PROMs of a gel formula containing a pool of amino acids and hyaluronic acid conceived for facilitating painless and fast wound closure in the oral cavity after simple single extraction. The null hypothesis was that there would be no differences in terms of PROMs and edema in the healing process in the two groups of treatment.

## 2. Materials and Methods

### 2.1. Sample Size Calculation

The sample size was estimated according to a previous published study with a similar design [12]. A minimum sample size of 40 subjects was required for to detect the possibility of a significant difference in PROMs (80% power, two-sided 5% significance level). A possible 5% drop-out rate was considered.

### 2.2. Subject Recruitment

This study was conducted in full accordance with the Declaration of Helsinki by the World Medical Association of 2008, updated in 2013. Each patient agreed and filled in the written informed consent to participate in the study (Ethical approval was granted in January/2021 by Saint Camillus International University of Health Sciences (UniCamillus, Rome), number 8/2020 (Protocol titled: “Studio clinico sull’efficacia del gel Aminogam nella terapia post-chirurgica di preservazione dell’alveolo estrattivo e nella guarigione dei tessuti molli.”). The patient cohort was composed of the patients coming to the Istituto Stomatologico Toscano in need for a single tooth extraction.

### 2.3. Inclusion Criteria

The main inclusion criterion was the patients’ need of a single tooth extraction, but not requiring antibiotic prophylaxis; thus, the authors named the surgery: “simple extraction”.

The other following inclusion criteria were adopted:

- Patients should be aged 18 years or older;
- Patients should exhibit good general health with no systemic disorders which might affect the healing phases (such as diabetes, cardiovascular events, immuno-depression);
- Patients do not require immediate rehabilitation (with an implant or other prosthetic technique) for the single edentulia before the 2 months of healing;
- Compliance with the study protocol and follow-up recall and willingness to adhere to the hygiene instructions and the use of a domiciliary gel.

### 2.4. Exclusion Criteria

Difficult extractions or wisdom tooth extractions that need antibiotics or corticosteroid therapy were excluded because this therapy may influence the healing phases and the relief of pain for patients [13].

The other exclusion criteria were: antibiotic prophylaxis for the dental extraction, alcohol or drug abuse, regular use of biphosphonate drugs, the use of steroidal anti-inflammatories, the use of anticoagulant drugs, the daily use of anti-acid therapy, the use of selective serotonin reuptake inhibitors, partaking in other therapies that might affect healing soft tissue phases, and smoking more than ten cigarettes; pregnancy; breastfeeding; previous periodontitis treatment within the last 6 months, radiotherapy to the head or neck region, current chemotherapy, and intra-operatory need to cut bone for the extraction.

Subjects exhibiting at least one of these criteria were excluded from the present study.

### 2.5. Study Design

This study was a randomized controlled clinical design with a 60-day follow-up. Eligible patients were enrolled from those attending the Tuscan Stomatologic Institute (Camaiore, Italy).

Eligible patients were equally randomized in two different groups of treatment (by a computer program generating the letter T or C) including:

- Test group (*n* = 20), who were not using the intra-operative hyaluronic acid-based gel (Polifarma Benessere S.r.l. & Professional Dietetics Aminogam^®^) (15 mL). After the procedure, patients received motivations and instructions for domiciliary maintenance using the same gel for 15 days, once a day in the evening after common oral hygiene procedures, which included brushing their teeth from the second day after surgery and using a chlorhexidine 0.20 mouthwash.
- Control group (*n* = 20), who were not using an intra-operative gel, nor were they performing domiciliary maintenance. These patients received motivations and instructions for common post-extractive oral hygiene procedures, which included brushing their teeth from the second day after surgery and using a chlorhexidine 0.20 mouthwash.

### 2.6. Surgical Procedures

Patients were prescribed a mouthwash with 0.2 chlorhexidine and were told to perform 2 rinses a day 5 days before the surgery if possible, according to the urgency of the extractive procedure. All patients rinsed their mouth for 1 min with 0.2 chlorhexidine mouthwash (Emoform^®^, Plak Out^®^ Active, Polifarma Benessere S.r.l., Rome, Italy) for decontamination purposes prior to the surgery (and twice a day for the following 2 weeks). Local anesthesia was performed using mepivacaine with adrenaline at 1:200,000. Tooth extraction was performed without raising a full thickness flap, and, if necessary, the tooth was sectioned to make the extraction the least traumatic possible. The surgeon treated the extraction socket with a 0.2 chlorhexidine rinse and then with a sponge of lyophilized non-denatured equine type-I collagen (Condress^®^, Smith & Nephew S.r.l., Monza, Italy) to the help the coagulum formation. Wounds were left to heal spontaneously without suturing.

### 2.7. Outcome Assessment

The primary outcome of the present study was the patients’ reported pain and swelling after dental extraction during the follow-up.

A VAS scale survey was administered to all patients immediately after the single extraction surgery, and at each follow-up.

The VAS scale was a questionnaire assessing the patients’ reported outcome concerning their pain and swelling perception with a numeric value from 0 up to 10.

In order to detect differences between test and control group in swelling and healing rate after the surgery, a 3D dental impression with an intra-oral scanner was performed for all patients before the surgery, immediately after, and at each follow-up. The measurement of each impression was defined as 100% at baseline (T0), simulating with the digital impression of the missing tooth. We then compared this starting volume to the volume of each scanning result in other follow-ups to evaluate the swelling and volume differences. A 3D intraoral scan was used for this purpose. All clinicians had previously received calibration for the accuracy of the use of the 3D dental impression with an intra-oral scanner during a 1-week training session (July 2020). A grid with reference points located at adjacent teeth was implemented to standardize the longitudinal analysis on the 3D scans.

The secondary outcome to assess the pain of patients is an indirect one: how many had taken painkillers and how often during the first 7 days after surgery.

Every complication or side-effect that occurred in the first 30 days after dental single extraction was annotated. The reasons for the extraction were reported.

### 2.8. Follow-Up Schedule

Baseline/To = immediately after single extraction;

T_1_ = 7 days after the extraction;

T_2_ = 14 days after the extraction;

T_3_ = 1 month after the extraction;

T_4_ = 2 months after the extraction.

### 2.9. Statistical Analysis

All parameters were expressed as mean ± standard deviation (SD). Student’s *t*-test was used for independent samples to calculate the difference between the test group and the control group. A value of *p* ≤ 0.05 was taken as statistically significant. Statistical analysis was performed using Excel (Microsoft, Windows 2020).

## 3. Results

A total of 40 patients with a mean age of 46.52 ± 9.84 years completed the 60-day follow-up after the surgery. The demographic data of both groups (test and control) are reported in Table 1. No significant differences were observed based on gender, age, or smoking habits (*p* value < 0.05) between the groups. No significant differences were observed between groups regarding the reported reason for the single extraction. A total of 40 single extractions were performed at baseline. A single tooth extraction per patient was included in the analysis.

**Figure 1:**
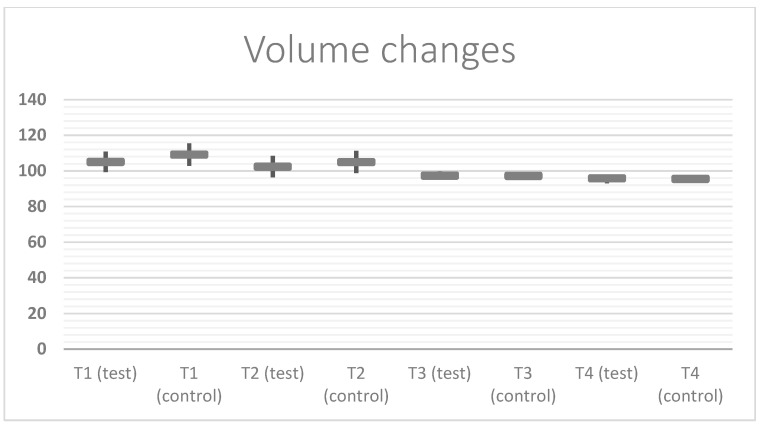
This is a figure showing the mean ± SD (standard deviation) of the volume changes from 100 % (baseline) of the soft tissue around the extracted tooth. The following scheme shows the statistically significant differences between the test and control groups at T_1_ (*p* value = 0.0380) and the slight differences at T_2_; the trend is similar at T_3_ and T_4_.

**Figure 2:**
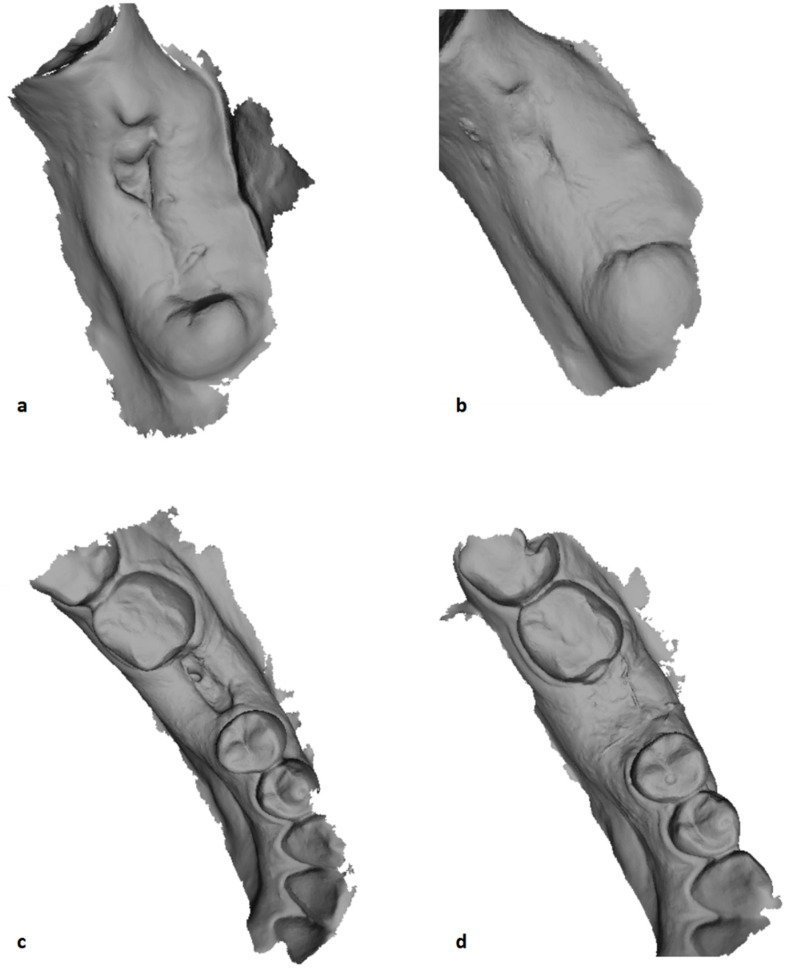
This is a figure showing two screenshot of the 3-D printing at the moment of volumetric calculation after 7 (T_1_: (**a**,**c**)) and 14 days (T_2_: (**b**,**d**)) from the first molar extraction in the test and control groups. (**a**) Post-extractive site at T_1_ in control group; (**b**) post-extractive site at T_2_ in control group. (**c**) Post-extractive site at T_1_ in test group; (**d**) post-extractive site at T_2_ in test group.

Mean and standard deviations for each outcome are reported in Table 2. All surgical sites healed uneventfully; in fact, all of the 3-D images showed complete healing at T_4_, and partial healing at T_1_. In particular, at the end of the follow-up period, the soft tissue healed completely, and no significant differences for volume changes could be highlighted by the 3-D intraoral scanning. Still, the edema changes in the test group, those using the home-care gel with amino acids, was significantly different and smaller at T_1_ than the control group, according to Student’s *t*-test (*p* value = 0.0380) (Figure 1 and Figure 2). The same happened with the pain parameter explored (VAS score) which was slightly higher in the control group at T_2_ and T_3_, but significantly (*p* value = 0.04) different at T_1_ (Table 2).

The VAS score significantly changed at each follow-up in the longitudinal interpatient analysis.

Even though no significant difference was found at T_2_, T_3_, and T_4_ between the groups for the experienced pain measured using the VAS scale, the total mean number of painkillers taken was 1.55 ± 1.10 units for the test group and 2.35 ± 1.18 units for the control group, with this effect being significant (*p* value 0.0327). Moreover, at T_1_, the consumption of painkillers was significantly different between the two groups, with 1.3 ± 0.98 units taken for the test group and 1.8 ± 1.10 units for the control group (*p* value = 0.0382).

## 4. Discussion

The present study was a randomized controlled clinical investigation, and it was based on patient-related outcomes concerning pain and the evaluation of the edema after tooth extraction with or without the post-operative use of a gel formula containing both HA and a pool of amino acids. All patients showed good healing with low to moderate pain and few complications. This may be due to the new trend of minimally invasive surgery that pushes clinicians to perform flapless extractions and without sutures; plus, the design of the study may have influenced the good results for the reported pain outcomes: only single extractions that did not require antibiotics or corticosteroid therapy in healthy patients were included in the study [14]. The amino acid and hyaluronic acid-based gel that we tested should enhance the endogenous process of cell reparation and defense, reducing the use of painkillers by patients and reducing the volume changes in the test group, meaning less swelling 1 week after the surgery.

The direct measure of pain (VAS) did not differ between groups, except for the first follow-up at 1 week, even though pain intensity decreased faster in the test group also at T_2_. The number of total painkillers taken was significantly different between groups, especially regarding the number used at T_1_. Those two measures of pain were more favorable in the test group. The first time point, one week after the extraction, is the most relevant in terms of the effectiveness of the amino acid-based gel. The surgical technique and the clinician’s experience in the present study may have shifted the outcomes to lower values of pain and swelling regardless of the gel use, but the surgeon’s skills might not always lead to these types of results in all clinical scenarios [15].

Plus, minimally invasive flapless surgery that involves applying a simple collagen sponge to the post-extractive site are recommended to reduce the swelling volume and facilitate the healing process, meaning that these surgical factors, such as not using high-pressure sutures, applying collagen sponges to the post-extractive socket, and performing a minimally invasive surgery that does not require antibiotic or corticosteroid therapy, also may have influenced the small volume changes reported in both groups [16,17].

The results of the present study regarding the reduction of the overall time of healing agreed with other clinical and in vitro studies [17,18]. It has been demonstrated that the investigated formula, containing high molecular weight sodium hyaluronate and four amino acids, which are precursors of collagen, increases the proliferative activity of fibroblasts, the synthesis of collagen I and III and fibronectin, the expression of interleukin 6 and 8 and connective tissue growth factor (CTGF), and the expression of vascular endothelial growth factor (VEGF) [18].

Collagen remodeling may be disturbed by a number of factors, leading to raised scar formation. This gel, thanks to its local administration and substantivity, promotes the deposition of components of the extracellular matrix and the production of cytokines, thus accelerating the wound healing process. Moreover, hyaluronic acid binds many water molecules, and so also improves tissue hydration and cellular resistance to mechanical damage [19,20].

Wound healing is a dynamic process that can be compromised in complex patients who are taking several drugs or with chronic and metabolic conditions. For this reason, some authors have associated the use of vitamin C and E with amino acid-enriched hyaluronic acid gel, reporting encouraging positive preliminary results on the healing of oral mucosa and on human gingival fibroblasts in vitro [21].

Other authors also focusing on the effect of the pool of amino acids combined to sodium hyaluronate in the same gel formula (Aminogam) agreed with the results of the present study [22,23].

The modality of the gel is based on human saliva, which relies on a similar mechanism to stimulate oral and skin primary wound closure, modulating the inflammatory response, especially in the early inflammation phase, which is involved in whole-wound healing. In addition, the properties of this gel, based on acid hyaluronic acid, also moderate the inflammatory response, which can contribute to the stabilization of the connective tissue matrix [24].

The absence of pressure due to sutures and the presence of a collagen sponge in a simple extraction with amino acids and hyaluronic acids might facilitate fast repair, reducing the risk of protracted detrimental activation of myofibroblasts, while the gel stimulates the initial proliferation and the action of fibroblasts and keratinocytes [25].

On the contrary other authors preferred to perform ridge preservation using xenograft biomaterials, whereby it is useful to maintain the bone and soft tissue volume [26,27,28].

In the post-extractive healing process, a non-resorbable materials could lead to volume maintenance, even though when the bone is completely healed there is still an artificial substance not defined as proper bone that it could lead to a worse prognosis in the case of infection.

In terms of the number of painkillers taken by patients, the group who used the test gel reported a lower intake of painkillers; this is in line with other studies that showed that hyaluronic acids also have pain-relieving properties [29,30].

Other methods have been proposed in the literature to increase the duration of the local action of hyaluronic acid in case of intraoral applications. However, adding four amino acids, such as in the hyaluronic acid-based gel tested in the present study, has highlighted an alternative method to increase the efficacy and effectiveness with a positive impact on the morbidity of oral surgery [31,32].

Even though the present study was performed with a control group of patients who were in good general health and who have not taken antibiotics, some variables on individual systemic conditions and the patients’ own oral health status could have influenced the results.

Reducing surgical iatrogenic factors in a controlled study with healthy patients could have mitigated the differences between the test and control group, reproducing clinical situations not so common in general dentistry. Moreover, other factors could have influenced the good results of both groups; for example, the use of more painkillers or even antibiotics or corticosteroids hidden to clinicians by patients.

## 5. Conclusions

Despite the limitations of the study regarding the type of surgery, the status of patients and the number of samples, the post-operative use of an amino acid and hyaluronic acid-based gel for the management of soft tissue healing after tooth extraction is a valid co-adjutant therapy to promote fast healing, reduce pain and volume changes, and allow patients to consume fewer painkillers, even without antibiotics or corticosteroid therapy. Additional studies with larger samples and a stratification of the population according to the type of extraction are required to support the results of the present study.

## Figures and Tables

**Table 1:** Anamnestic data of two treatment groups of treatment; SD: standard deviation, N°: “number of”.

Treatment Group	Test	Control
N patients	20	20
Female	11	9
Male	10	10
Age (mean ± SD)	45.30 ± 10.00	47.75 ± 10.00
Upper teeth extracted	10	9
Lower teeth extracted	10	11
Second premolars	8	9
First molars	9	10
Second molars	3	1
Tooth extracted for mobility	6	4
Tooth extracted for fracture	12	13
Tooth extracted for a failed endodontic treatment	2	3
N° patients smoking (<6 cigarettes)	3	2

**Table 2:** For each group, volume (baseline 100%), VAS score (0–10), and number of painkillers.

Volume (Baseline: 100%) Mean Value ± SD (Standard Deviation)	T_0_	T_1_	T_2_	T_3_	T_4_
Test (N patients = 20)	100% ± 0.00	105.05% ± 5.74	102.35% ± 6.05	97.35% ± 2.34	95.85% ± 1.81
Control (N patients = 20)	100% ± 0.00	109.15% ± 6.30	104.95% ± 6.26	97.20% ± 2.26	95.55% ± 1.88
*p* value		0.0380	0.1895	0.1895	0.8380
VAS score (0–10) Mean value ± SD (standard deviation)	T_0_	T_1_	T_2_	T_3_	T_4_
Test (N patients = 20)	3.9 ± 2.02	1.65 ± 0.75	1.1 ± 0.91	0.1 ± 0.31	0 ± 0
Control (N patients = 20)	4.5 ± 2.06	2.7 ± 1.49	1.35 ± 1.04	0.2 ± 0.41	0 ± 0
*p* value	0.82	0.04	0.86	0.94	/
Number of painkillers taken Mean value ± SD (standard deviation)	T_0_	T_1_	T_2_	T_3_	Total
Test (N patients = 20)	1.1 ± 1.24	1.3 ± 0.98	0.25 ± 0.44	0 ± 0	1.55 ± 1.10
Control (N patients = 20)	0.95 ± 1.05	1.8 ± 1.10	0.55 ± 0.60	0 ± 0	2.35 ± 1.18
*p* value	0.49	0.0382	0.1735	/	0.0327

## Data Availability

Additional data may be available if requested from the authors at Tuscan Stomatologic Institute. The data are not publicly available because the clinical study is still on-going with a longer follow-up.
